# Subnormothermic Perfusion in the Isolated Rat Liver Preserves the Antioxidant Glutathione and Enhances the Function of the Ubiquitin Proteasome System

**DOI:** 10.1155/2016/9324692

**Published:** 2016-10-09

**Authors:** Teresa Carbonell, Norma Alva, Sergio Sanchez-Nuño, Shannamar Dewey, Aldrin V. Gomes

**Affiliations:** ^1^Department of Cell Biology, Physiology and Immunology, University of Barcelona, Avda Diagonal 643, 08028 Barcelona, Spain; ^2^Department of Physiology, Neurobiology and Behavior, University of California, Davis, 176 Briggs Hall, One Shields Avenue, Davis, CA 95616, USA

## Abstract

The reduction of oxidative stress is suggested to be one of the main mechanisms to explain the benefits of subnormothermic perfusion against ischemic liver damage. In this study we investigated the early cellular mechanisms induced in isolated rat livers after 15 min perfusion at temperatures ranging from normothermia (37°C) to subnormothermia (26°C and 22°C). Subnormothermic perfusion was found to maintain hepatic viability. Perfusion at 22°C raised reduced glutathione levels and the activity of glutathione reductase; however, lipid and protein oxidation still occurred as determined by malondialdehyde, 4-hydroxynonenal-protein adducts, and advanced oxidation protein products. In livers perfused at 22°C the lysosomal and ubiquitin proteasome system (UPS) were both activated. The 26S chymotrypsin-like (*β*5) proteasome activity was significantly increased in the 26°C (46%) and 22°C (42%) groups. The increased proteasome activity may be due to increased Rpt6 Ser120 phosphorylation, which is known to enhance 26S proteasome activity. Together, our results indicate that the early events produced by subnormothermic perfusion in the liver can induce oxidative stress concomitantly with antioxidant glutathione preservation and enhanced function of the lysosomal and UPS systems. Thus, a brief hypothermia could trigger antioxidant mechanisms and may be functioning as a preconditioning stimulus.

## 1. Introduction

A major clinical problem in liver surgery and transplantation is damage due to transient ischemia and reperfusion [1–3]. The pathological process involves a variety of cellular dysfunctions caused by increased production of reactive oxygen species and inflammatory responses [4]. Several studies suggest that the induction of mild (32–35°C) to moderate (28–32°C) hypothermia may attenuate the progression of liver damage against ischemia [1, 5, 6]. Moreover, perfusion in* ex vivo* machines resulted in improved viability at subnormothermic temperatures (20-21°C), in both livers from experimental animals [7–11] and from humans [12, 13]. These authors also found that many livers which would be discarded due to their low quality could be rescued for transplantation thanks to the restorative effects of subnormothermic perfusion.

There is growing interest in the use of hypothermia to prevent ischemic damage in clinical and experimental trials [14]. The protective effects of hypothermia are suggested to primarily be a consequence of decreased cellular metabolism, thus conserving ATP levels [15]. This proposal implies a passive method for hypothermia-induced protection and does not explain the wide role of therapeutic hypothermia against many injuries and in different tissues. Reduction of oxidative stress, as observed in ischemic cardiomyocytes [16], and attenuation in the consumption of endogenous antioxidants, as seen in hypoxic brains [17, 18], could be two additional mechanisms.

In the liver, the observed hepatoprotective effects of hypothermia against ischemia include prolonging survival, attenuation of liver damage [1, 5], suppressed reactive oxygen species, and improved sinusoidal perfusion [19, 20]. Mild hypothermia also attenuated the progression of liver injury induced by other agents, such as acetaminophen in mice [21] and hepatocarcinogenesis in rats [22].

Cellular pathways affected by hypothermia have been reviewed by our group and others [23–25]. Most of the studies cited in these reviews focused on mechanisms related to inflammation, free radicals, or apoptosis, and consistently demonstrated that when hypothermia is induced before ischemic, hypoxic, or toxic episodes, it is able to reduce deleterious pathways while enhancing protective events.

These effects of hypothermia lead us to consider whether hypothermia could have a more active role in preventing damage through triggering antioxidative mechanisms of cell protection. Given the recent contributions that perfusion of isolated livers at subnormothermic temperature protects cellular integrity [9, 12], we investigated isolated perfused rat livers (IPRL) at a range of temperatures from normothermia (37°C) to subnormothermia (26°C and 22°C) for a brief period of time (15 minutes) and then investigated oxidative damage parameters and antioxidant defenses. Jung et al. [26] identified three lines of defense against oxidative stress: the first includes antioxidant molecules (such as glutathione), the second includes enzymatic antioxidants, and the third involves repair system proteins, including the proteolytic pathways. Of the two systems of intracellular proteolysis, the lysosomal system plays an important role in the degradation of membrane-bound proteins while the ubiquitin proteasome system (UPS) is widely recognized as the main system for degradation of cytosolic proteins [27, 28]. Our results suggest hypothermia limits ischemic damage by activating protective mechanisms in two of the oxidative stress defensive categories: antioxidant molecules and repair system proteins. We detected increased glutathione levels and proteolytic activity which may limit ischemia induced oxidative damage.

## 2. Materials and Methods

### 2.1. Animals and Liver Isolation and Perfusion

Adult male Sprague-Dawley rats (225–250 g body weight) were used in this study. Rats were fasted overnight and had free access to water. Rats were anesthetized with i.p. sodium pentobarbital (65 mg/Kg). Heart failure was induced by incision in the diaphragm and the liver was isolated and connected for perfusion in a nonrecirculating IPRL system at a flow rate of 3 mL/min/g liver with Krebs-Henseleit buffer (KHB) (mM): 118 NaCl, 4.7 KCl, 1.2 MgSO_4_, 1.2 KH_2_PO_4_, 2.5 CaCl_2_, 25 NaHCO_3_, 20 Hepes (pH 7.4), aerated with 95% O_2_, and 5% CO_2_ [29]. The procedure was approved by the University of Barcelona Institutional Committee of Animal Care and Research and followed European Community guidelines.

Livers were perfused at 37°C for 15 min to stabilize. Then they were randomly distributed in the three experimental groups that were perfused at 37°C, 26°C, or 22°C for 15 min (total time of experiment lasting 30 min). As an index of cellular injury, plasma alanine aminotransferase (ALT) was measured in the effluent using a commercial kit (BioSystems, Barcelona, Spain). After perfusion, livers were frozen in liquid nitrogen and stored at −80°C until analysis.

Nitric oxide (NO) regulates the hepatic microvascular perfusion through its vasodilatory effect and through its anti-inflammatory actions [30]. NO levels were measured as nitrate plus nitrite in liver homogenates in 10% (w/v) PBS, centrifuged at 2000 ×g for 5 min, and ultrafiltered by means of a 30 kDa molecular weight cut-off filter. In the assay, nitrate was converted to nitrite using nitrate reductase and total nitrite was measured using the Griess reaction and a colorimetric assay kit (Cayman Chemical Co., Ann Arbor, MI, USA) and expressed as nmol/mg protein.

All other chemicals were purchased from Sigma-Aldrich Chemical.

### 2.2. Oxidant Assays

Lipid peroxidation in the liver was determined as the end product malondialdehyde (MDA) by thiobarbituric reactive substances (TBARS) assay [31]. Liver was homogenized with a teflon bar in 10% (w/v) RIPA solution, (Tris 50 mM pH 7.4, 1% Triton X-100, NaCl 150 mM, NaF 5 mM, 0.1% sodium dodecyl sulphate, and 1% sodium deoxycholate) with antiprotease solution (aprotinin at 1.7 mg/mL, 2 *μ*g/mL pepstatin, 2 *μ*g/mL leupeptin and 1 mM phenylmethylsulfonyl fluoride, and sodium orthovanadate at 1 mM). The suspension was centrifuged at 2000 ×g for 5 min and the pellet discarded. The formation of MDA-TBA adduct was fluorometrically measured at an excitation wavelength of 515 nm and an emission wavelength of 550 nm. The calibration curve was determined using tetraethoxypropane. Values are expressed as TBARS in nmol/mg protein.

Advanced oxidation protein products (AOPP) have been identified as a biomarker of oxidative damage to proteins, detecting dityrosine-containing and cross-linking protein products, but also a novel mediator of inflammation [32]. AOPP in liver homogenates were assayed by a modification of Witko–Sarsat's method [32, 33]. The formation of AOPP was spectrophotometrically measured at 340 nm and results were obtained through a standard calibration curve using 100 *μ*L of chloramine-T solution (0–100 *μ*mol/L). AOPP concentration was expressed as *μ*mol/L of chloramine-T equivalents.

### 2.3. Total Protein Determination

The total liver proteins were determined using the Bradford protein assay [34].

### 2.4. Antioxidant Assays

Reduced (GSH) and oxidized glutathione (GSSG) were measured in the liver extracts using the procedure described by Hissin and Hilf [35] and modified by Alva et al. [36]. Tissue was homogenized in cold buffer containing 5 mm phosphate-EDTA buffer (pH 8.0) and 25% HPO_3_. The homogenates were ultracentrifuged at 100,000 ×g and 4°C for 30 min, and the resulting supernatant was used to determine GSH and GSSG concentrations, using the fluorescent probe* o*-phthalaldehyde. Fluorescence was determined at a wavelength emission of 420 nm and excitation at 350 nm.

Glutathione reductase (GSH-R, EC 1.6.4.2) is a flavoprotein that catalyzes the NADPH-dependent reduction of oxidized glutathione (GSSG) to reduced glutathione (GSH). For GSH-R determination, tissue was homogenized in cold buffer (50 mM potassium phosphate, pH 7.5, 1 mM EDTA) and centrifuged at 10,000 ×g for 15 min (4°C). The resulting supernatant was used to measure GSH-R activity with Cayman Chemical Glutathione Reductase Assay Kit (Cayman Chemical Co., Ann Arbor, MI, USA) by measuring the rate of NADPH oxidation. The oxidation of NADPH to NADP+ is accompanied by a decrease in absorbance at 340 nm. Results of GSH-R activity are expressed as mU/mg protein.

### 2.5. Proteolytic Activity Measurement of Cathepsin B and Cathepsin L and 26S Proteasome

Proteolytic activities were measured as previously described by our laboratory [37–39]. Cathepsin B and Cathepsin L assays were carried out in a total volume of 100 *μ*L per well in black 96-well plates. For the cathepsin B activity, protein samples (25 *μ*g) were incubated with 100 *μ*M substrate Z-Arg-Arg-AMC (Biomol) in 44 mM potassium phosphate buffer, 6 mM sodium phosphate, 0.67 mM EDTA, and 1.35 mM cysteine (pH 6.0). For cathepsin L activity, protein samples (25 *μ*g) were incubated with 100 *μ*M substrate Z-Phe-Arg-AMC (Peptides International, Louisville, KY, USA) in 100 mM sodium acetate buffer, with 1 mM EDTA and 1 mM DTT (pH 5.5). The assay was conducted in the absence and presence of a specific inhibitor to determine specific activity: for cathepsin B, 10 *μ*M CA-074 (Enzo, Life Sciences, Farmingdale, NY, USA) was used, and to inhibit cathepsin L, 10 *μ*M Cathepsin L inhibitor I (Calbiochem, La Jolla, CA, USA) was used. Released AMC was measured using a Fluoroskan Ascent fluorometer (Thermo Electron) at 390 nm (excitation wavelength) and 460 nm (emission wavelength) for up to 120 min.

The ATP-dependent 26S proteasome activities were measured in the presence of 0.1 mM ATP [40]. Liver cell lysates were prepared by homogenization in 50 mM Tris, 1 mM EDTA, 5 mM MgCl_2_, 150 mM NaCl, and 1 mM DTT, pH 7.5. The samples were then centrifuged at 12,000 ×g for 15 min (4°C) and the supernatants were collected. To analyze the 26S proteasome activity of liver homogenates (24 *μ*g/sample), the fluorescent substrates Z-LLE-AMC 100 *μ*M for *β*1, Boc-LSTR-AMC for *β*2, and Suc-LLVY-AMC 100 *μ*M for *β*5 were used. Each assay was conducted in the absence and presence of a specific proteasome inhibitor Z-Pro-Nle-Asp-H (Enzo) 40 *μ*M for *β*1, epoxomicin (Peptides International) 40 *μ*M for *β*2, and epoxomicin 10 *μ*M for *β*5 to determine proteasome-specific activity. All the assays were carried out in a total volume of 100 *μ*L. Released AMC was measured using a Fluoroskan Ascent fluorometer (Thermo Electron) at an excitation wavelength of 390 nm and an emission wavelength of 460 nm.

### 2.6. Western Blot Analysis and Quantification

Whole liver cell lysates were prepared by homogenizing the livers in: 50 mM Tris (pH 7.5), 1 mM EDTA, 5 mM MgCl_2_, 150 mM NaCl, supplemented with DTT, and protease inhibitors (final concentrations were 10 *μ*g/mL aprotinin, 2 *μ*g/mL pepstatin, 2 *μ*g/mL leupeptin, and 1 mM phenylmethylsulfonyl fluoride). The samples were then centrifuged at 12,000 ×g for 10 min and the protein concentration measured in the supernatant. Supernatants were treated with Laemmli loading buffer and 50 *μ*g of proteins resolved on SDS-polyacrylamide (10%) gels and transferred to nitrocellulose. Membranes were then blocked for 1 h with 3% nonfat dry milk (NFM) in Tris-buffered saline (TBS) (pH 7.4) containing 0.05% (w/v) Tween 20 (TTBS). The membranes were washed three times in TTBS and probed overnight with the following primary antibodies: anti-4-HNE conjugates (Novus Biologicals, Littleton, CO, USA), anti-ubiquitin (Sigma-Aldrich), anti-Rpt6, phospho S120 Rpt6 (affinity purified rabbit antibody commercially made by 21st Century Biochemicals), anti-Rpt1, and 20S core subunits (*α*5/*α*7, *β*1, *β*5, *β*5i, and *β*7) (Enzo life sciences). Detection was performed with anti-IgG-HRP (Santa Cruz Biotechnology, Inc., Heidelberg, Germany). The blots were visualized with enhanced chemiluminescence (SuperSignal West Pico Chemiluminescent Substrate, Thermo Scientific, Rockford, IL, USA) and detected and scanned on Fujifilm LAS-3000 Imager (Fujifilm Corporation, Tokyo, Japan). Digital images were quantified using Quantity One software (Bio-Rad, Hercules, CA, USA) and normalized to *β*-actin (Sigma-Aldrich, MO, USA). Ponceau S staining as a loading control was used for anti-Rpt6 and phospho S120 Rpt6 western blots due to high protein load (80 *μ*g) [41].

### 2.7. Statistical Analysis

Results are expressed as means ± SEM of six animals. Data were processed using the statistical software GraphPad InStat (GraphPad Software, Inc., San Diego, CA, USA). The means of the experimental groups were analyzed by two-way ANOVA using the Student–Newman–Keuls test to identify significant differences (when *P* < 0.05) between the groups.

## 3. Results

### 3.1. Subnormothermic Perfusion Preserves Liver Function but Increases Oxidative Stress and Protein Oxidation

**Figure 1:**
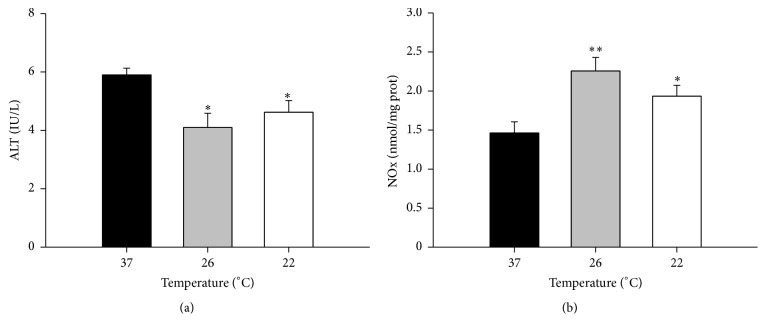
Temperature effects on the hepatic function of isolated perfused rat (IPR) livers. IPR livers were perfused in a nonrecirculating system with Krebs-Henseleit buffer at 37°C for 15 min, for stabilization, and then at the indicated temperatures for 15 min more. Results show cell viability by ALT assay in the perfusate (a) and NO production in liver homogenates (b). Values expressed as mean ± SEM of *n* = 6. Significant differences from livers perfused at 37°C: ^*∗*^ *P* < 0.05 and ^*∗∗*^ *P* < 0.01.

**Figure 2:**
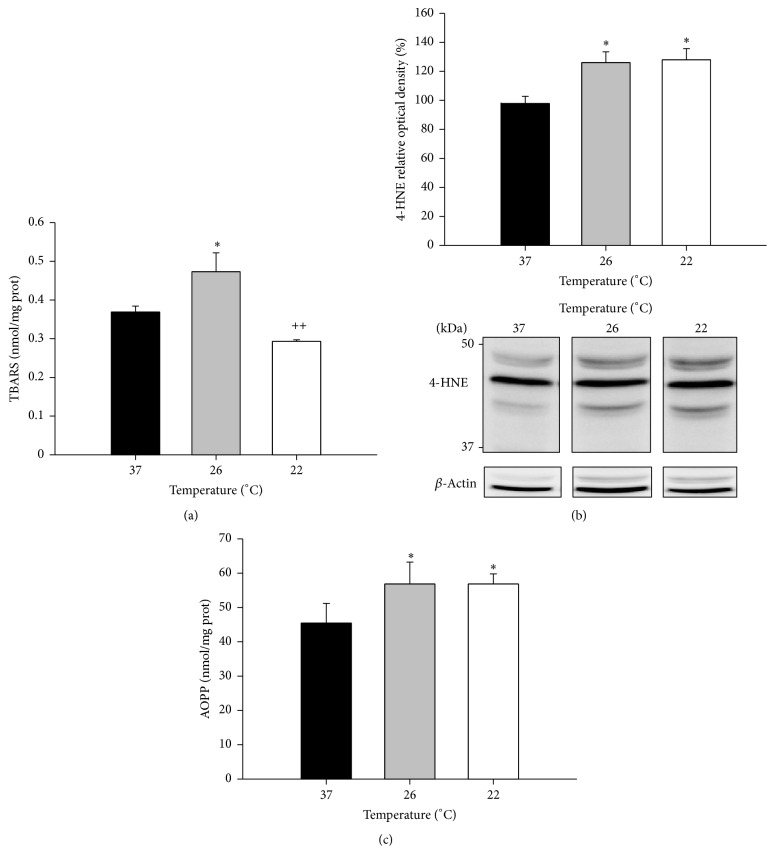
Lipid and protein oxidation in isolated perfused rat livers. IPR livers were perfused in a nonrecirculating system with Krebs-Henseleit buffer at 37°C for 15 min, for stabilisation, and then at the indicated temperatures for 15 min more. Lipid peroxidation was measured as TBARS (a) and by western blotting of HNE-protein adducts (b), and advanced oxidation protein products (dityrosine-containing and cross-linking protein products) measured spectrophotometrically at 340 nm (c). Values expressed as mean ± SEM of *n* = 6. Significant differences from livers perfused at 37°C: ^*∗*^ *P* < 0.05. Significant differences from livers perfused at 26°C: ^++^ *P* < 0.01.

Our results showed that subnormothermic perfusion preserves liver integrity and function, as reflected by decreased ALT levels in the perfusate (Figure 1(a)), and increased NO in liver (Figure 1(b)). Because NO has an extremely short half-life, we measured it indirectly by quantifying the final products of its reaction, nitrates, and nitrites. These levels increased from 1.48 ± 0.14 at 37°C to 2.27 ± 0.17 at 26°C (*P* < 0.01) and 1.945 ± 0.14 at 22°C (*P* < 0.05). However subnormothermia resulted in higher levels of oxidative stress markers and protein oxidation. One of the effects of oxidative stress is lipid peroxidation which involves the interaction of free radicals with polyunsaturated fatty acids. The end products of lipid peroxidation are reactive aldehydes, such as malondialdehyde (MDA) measured as thiobarbituric reactive substances (TBARS) and 4-hydroxynonenal (HNE). Proteins are particularly susceptible to changes caused by HNE, and HNE-protein adducts formation plays a significant role in many cellular processes [42]. We have found increased levels of TBARS after subnormothermic perfusion at 26°C (*P* < 0.05) (Figure 2(a)) and increased HNE-protein adducts at both subnormothermic perfusion temperatures (*P* < 0.05) (Figure 2(b)). Oxidation induces several modifications in proteins that can lead to new compounds and modified structures. AOPP, which measures dityrosine-containing and cross-linking protein products, increased by 27% in livers from both subnormothermic perfusion ranges (*P* < 0.05) (Figure 2(c)).

### 3.2. Subnormothermic Perfusion and Antioxidant Status

**Figure 3:**
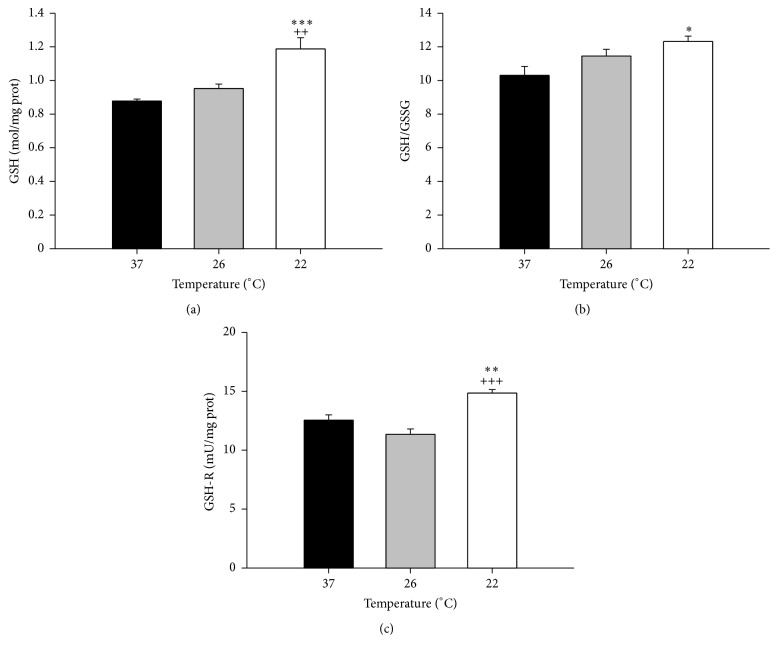
Temperature effects on antioxidant status in isolated perfused rat livers. IPR livers were perfused at 37°C for 15 min and then at the indicated temperatures for 15 min more. Bar graphs show hepatic GSH (a), GSH/GSSG ratio (b), and GSH-reductase activity (c). Values expressed as mean ± SEM of *n* = 6. Significant differences from livers perfused at 37°C: ^*∗*^ *P* < 0.05, ^*∗∗*^ *P* < 0.01, and ^*∗∗∗*^ *P* < 0.001. Significant differences from livers perfused at 26°C: ^++^ *P* < 0.01 and ^+++^ *P* < 0.001.

Previous results show that the hepatic GSH [36] and the hepatic reduced/oxidized glutathione ratio (GSH/GSSG) increased in hypothermic rats [43] and in* ex vivo* subnormothermic rat liver [9]. Therefore, we measured the levels of oxidized and reduced glutathione and the activities of the enzyme responsible for the formation of reduced glutathione, glutathione reductase (GSH-R). No differences were observed in GSH levels, GSH/GSSG ratio, and GSH-R activity at 26°C (Figures 3(a), 3(b), and 3(c)). However, at 22°C GSH and the GSH/GSSG ratio rised, and the activity of GSH-R increased by 18%.

### 3.3. Subnormothermic Perfusion Increased the Activity of Cathepsin B and Cathepsin L

**Figure 4:**
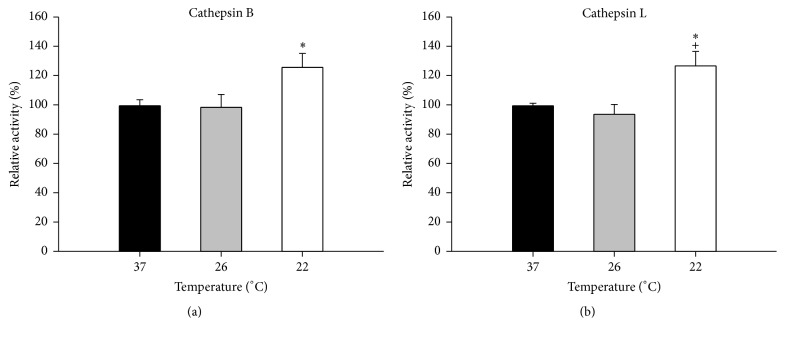
Lysosomal activity in isolated perfused rat livers. IPR livers were perfused at 37°C for 15 min and then at the indicated temperatures for 15 min more. To independent lysosomal enzymes, cathepsin B (a) and cathepsin L (b) were measured to determine if lysosomal activity was affected. Data is expressed as mean ± SEM of *n* = 6. Significant differences from livers perfused at 37°C: ^*∗*^ *P* < 0.05. Significant differences from livers perfused at 26°C: ^+^ *P* < 0.05.

The largest group of hydrolases in lysosomal compartments are the cathepsin proteases. Investigation of two cathepsins, B and L, showed that the enzymatic activity of these proteases increased by 26-27% in subnormothermic livers perfused at 22°C (*P* < 0.05) (Figures 4(a) and 4(b)) compared to the normothermic group (37°C) suggesting that the lysosomal activity is increased.

### 3.4. Subnormothermic Perfusion Increased Ubiquitinated Proteins and the Activity of 26S Proteasomes

**Figure 5:**
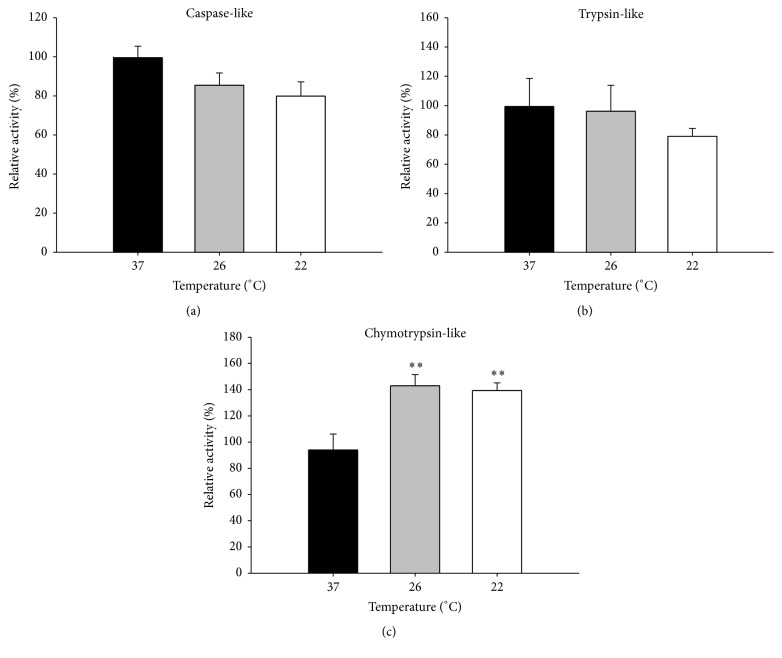
Proteasomal activity of 26S proteasome in isolated perfused rat livers. IPR livers were perfused at 37°C for 15 min and then at the indicated temperatures for 15 min more. The caspase-like *β*1 (a), trypsin-like *β*2 (b), and chymotrypsin-like *β*5 (c) activities of the proteasome were determined. Data is expressed as mean ± SEM of *n* = 6. Significant differences from livers perfused at 37°C: ^*∗∗*^ *P* < 0.01.

**Figure 6:**
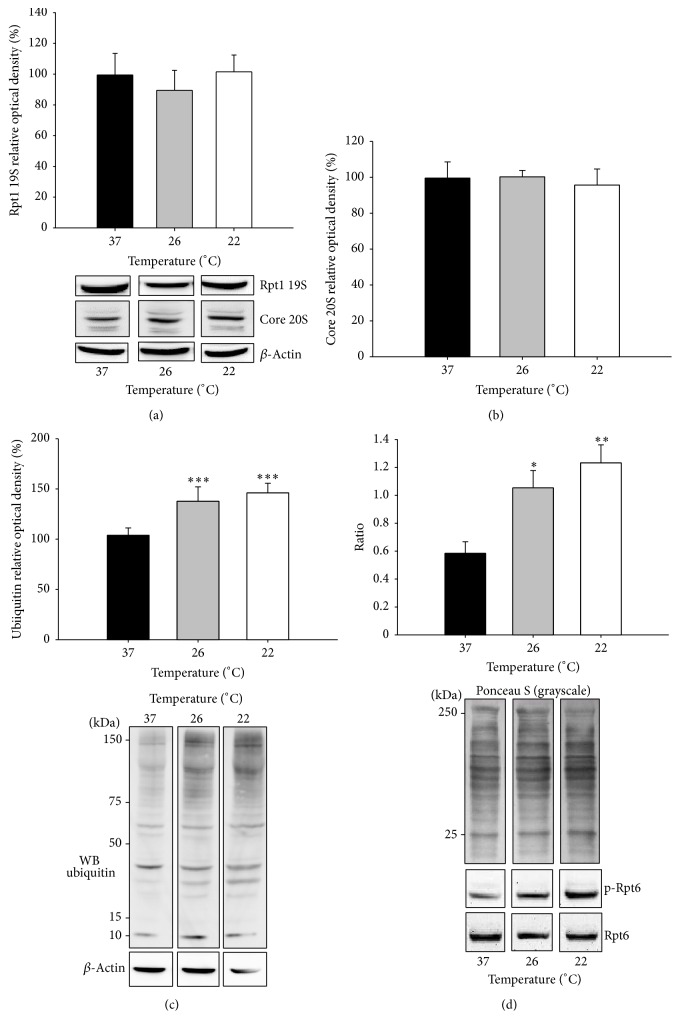
Expression of ubiquitin proteasome system in isolated perfused rat livers. IPR livers were perfused at 37°C for 15 min and then at the indicated temperatures for 15 min more. Results show western blots and densitometric analysis for Rpt1 19S (a) and 20S core subunit (b). (c) Levels of polyubiquitinated proteins; densitometry includes all polyubiquitinated bands detected. *β*-Actin was used as a normalization control for the western blots. (d) Ponceau S (total protein) staining was used as loading control for phospho S120 Rpt6 and Rpt6 densitometric analyses of western blots; values were then used to calculate p-Rpt6/Rpt6 ratios. Results are expressed as mean ± SEM of *n* = 3 to 6 independent samples per group. Significant differences from livers perfused at 37°C: ^*∗*^ *P* < 0.05, ^*∗∗*^ *P* < 0.01, and ^*∗∗∗*^ *P* < 0.001.

Investigation of the three proteolytic activities of the proteasome, the caspase-like *β*1, trypsin-like *β*2, and chymotrypsin-like *β*5 activities, indicated that proteasome activity was increased. While the 26S (ATP-dependent) caspase-like and trypsin-like activities were not significantly affected by the subnormothermic perfusion (Figures 5(a) and 5(b)), the chymotrypsin-like proteasome activity was significantly increased at 26°C (46%) and at 22°C (42%) (*P* < 0.01) (Figure 5(c)). This is especially interesting since the expression of several 20S core subunits (Figure 6(b)) and the expression of Rpt1 ATPase of the 19S proteasome subunit (Figure 6(a)) were not affected by subnormothermic perfusion. These results suggest that the increased proteasome activity may be due to posttranslational modifications or changes in the levels of associating partners on the proteasomes rather than its expression. We have previously shown that proteasomes from different tissues have different levels of associating partners which can affect proteasome activity [39].

To determine if the levels of polyubiquitinated proteins are affected by subnormothermic perfusion we utilized western blotting and observed that subnormothermic perfusion resulted in increased levels of polyubiquitinated proteins (Figure 6(c), *P* < 0.001). Since ubiquitinated proteins are selectively degraded by the 26S proteasome and Rpt6 S120 phosphorylation has been shown to increase 26S proteasome activity [44], the levels of Rpt6 S120 phosphorylation were investigated. A significant increase in the ratio of phospho-Rpt6/Rpt6-ATPase of the 19S proteasome subunit, from 0.59 ± 0.06 at normothermia to 1.06 ± 0.09 (*P* < 0.05) at 26°C and 1.24 ± 0.1 (*P* < 0.01) at 22°C (Figure 6(d)), was detected. These results suggest that the increased activity of the proteasome that occurs with subnormothermic perfusion may be due to increased phosphorylation of the serine 120 subunit of Rpt6.

## 4. Discussion

There is widespread consensus that treatment with hypothermia protects against various types of damage in many organs [25, 45–47]. Even so, the early events triggered by hypothermic treatment are not yet well defined. Potential mechanisms suggested for such protection include reduction of oxidative stress [16], increased endogenous antioxidants [17, 18], reduction of inflammatory mediators [48], and decreased apoptosis [49]. These effects of hypothermia, together with the improved viability seen in subnormothermic perfused livers [7, 9, 12], lead us to consider whether perfusion at subnormothermic temperatures for a limited period of time could trigger antioxidant mechanisms that confer cellular protection.

The present study demonstrated that liver integrity was retained at subnormothermic temperatures despite increased lipid and protein oxidation levels. Preserved cellular integrity was reflected by low ALT levels in the perfusate and increased NO in the liver. Increased synthesis of NO has been shown to be protective, as it reduces neutrophil adhesion and platelet aggregation while preventing microcirculatory disturbances in liver blood flow [50, 51]. NO has also been proposed to play a key role in both initiating and mediating ischemic preconditioning [30] and is involved in the preconditioning response for ischemia-reperfusion injury in fatty livers [50, 52]. Despite these protective effects, we also measured increased oxidation levels: lipid peroxidation, such as malondialdehyde MDA (measured as TBARS) and protein oxidation (measured by AOPP and HNE-protein adducts) were increased in subnormothermic perfused livers.

Although some results showed that in liver the protein expression of antioxidant enzymes and their activity were relatively unaffected by one hour of cold exposure in rats [53] and in hibernating animals [54], the GSH reductase activity was previously shown to significantly increase after cold exposure in rat livers [55]. Furthermore, the hepatic GSH [36] and reduced/oxidized glutathione ratio (GSH/GSSG) [9, 43] were shown to increase after one to six hours at subnormothermic temperature in rats. However, the early effects of brief exposure to subnormothermic temperature on antioxidant enzymes have not been previously reported. In the present work we found increased levels of GSH, the GSH/GSSG ratio, and the activity of GSH-R in livers perfused at 22°C during 15 min. Although increased levels of GSH and GSH-R activity suggest increased antioxidant capability of the liver, the increased levels of lipid and protein oxidation suggest that the higher antioxidant capacity is at least initially unable to completely prevent lipid peroxidation and the resulting protein damage.

The lipid peroxidation process is a chain reaction that produces multiple breakdown molecules, such as the aldehydes MDA and 4-HNE [56]. 4-HNE appears to be the most toxic product of lipid peroxidation [57]. 4-HNE can modify proteins by forming covalent adducts, accelerating protein aggregation [42]. Oxidative modifications of proteins can affect their physiological activity and typically increase their degradation rate. Several reports have highlighted that mild oxidation of proteins increases their susceptibility to proteasomal degradation [58, 59]. The excessive accumulation of such abnormal proteins can exacerbate the apoptotic or necrotic pathways in the cell [60]. As such, removing abnormal proteins may prevent oxidative stress induced cell death. This function, largely due to the proteasome, has been suggested to be part of the antioxidant defense [26].

The mechanisms by which lipid peroxidation modified proteins are removed from the cell have been studied in different models. 4-HNE exposure increases ubiquitination of adiponectin [61] and the UPS was shown to be the predominant proteolytic enzyme involved in the removal of 4-HNE adducts of alcohol dehydrogenase [62]. In kidney homogenates, HNE-modified proteins were shown to be specifically degraded by the UPS [63]. However, in lens epithelial cells, HNE-modified proteins are ubiquitinated but degraded by the ubiquitin-dependent lysosomal pathway rather than by the proteasome [64]. Moreover, electron micrographs of 4-HNE-treated cells showed extensive vacuolization and treatment with lysosomal inhibitors induced cell death. These findings suggest that lipid peroxidation-derived aldehydes also stimulate autophagy [65]. The present study implicates elevated activity in both the UPS and lysosomal pathways.

The lysosome system in the liver is involved in the degradation of membrane-bound or organelle-associated proteins and aggregates. The largest group of hydrolases in lysosomal compartments are the cathepsin proteases, essential for the proteolysis of protein substrates [66]. Cathepsins are responsible for intracellular degradation of Advanced Glycation End Products- (AGEs-) modified proteins [67] and following oral administration of AGEs, upregulation of cathepsins B and L activities was reported in* Drosophila melanogaster* [68]. In our model, isolated liver perfusion at 22°C (but not at 26°C) resulted in increased activity of the two lysosomal proteases investigated (cathepsins B and L) when compared to control temperatures (37°C). This increased lysosomal activity may be important in limiting lipid peroxidation damage and removing some of the oxidized proteins (HNE-protein adducts and AOPP). Increased lysosomal activity (lysosomal degradation of autophagosomes) has been shown to protect cardiac myocytes against ischemia/reperfusion injury [69]. Hence it is possible that the increased lysosomal activity at 22°C may be protective against ischemia/reperfusion injury.

Oxidized proteins can also be removed by the proteasome [37, 59] via ubiquitin independent degradation as well as ubiquitin tagging and subsequent targeting by the 26S proteasome [28]. Conversely, it has been suggested that an excess of HNE may directly form adducts on the three proteolytic subunits of the proteasome, thereby reducing its enzymatic activity and contributing to the accumulation of modified proteins [70]. Oxidative damage to various subunits of the 26S proteasome during ischemia and reperfusion has also been reported [60]. Seemingly in support of this data, we observed higher levels of polyubiquitinated proteins at 26°C and 22°C when compared to 37°C. Similarly, ubiquitin conjugated proteins increased 2-3-fold during torpor in the liver of hibernating squirrels [71].

The intact 26S proteasome is composed of one or two 19S regulatory particles at each end of a cylindrical 20S core particle. Each protein particle has a complex quaternary structure consisting of many (19–28) subunits. Degradation of protein substrates requires the 19S particle to recognize the ubiquitin-conjugated protein and regulate the entry of the substrate in the proteolytic cavity of the 20S core particle. The expression of several 20S and 19S subunits of the proteasome was not affected by subnormothermic temperature in our study, indicating no effect on the number of proteasomes assembled. However, we found a significant increase in the phosphorylation of the 19S subunit Rpt6 at serine 120. Previous studies have revealed that phosphorylation of proteasome subunit Rpt6 increases 26S proteasome activity and that Rpt6 phosphorylation may be an important regulatory mechanism for proteasome-dependent control [44, 72, 73]. Covalent regulation via phosphorylation allows for a quick increase in the activity of the UPS independent of increasing the overall number of proteasomes. Consistent with an increase in the levels of phosphorylated Rpt6, the 26S chymotrypsin-like activity of the proteasome was significantly increased in livers perfused at both 26°C and 22°C subnormothermic temperatures. Considering these results and the findings that ischemic preconditioning also enhances proteasome function [74], it seems probable that the increased proteasome activity is protective. Additionally, elevated proteasome activity has been shown to be associated with long-lived humans (centenarians) [75] and very long-lived animals such as the naked mole rat [76].

## 5. Conclusions

Overall, our results suggest that the early events initiated by hypothermia include the induction of oxidative stress and concurrent stimulation of several potential mechanisms of cell protection, including increased NO and GSH levels and the activation of lysosomal and proteasomal systems to repair oxidative damage. The increased proteasomal activity is likely due, in part, to phosphorylation of serine 120 on the proteasome Rpt6 subunit. Hypothermia may be acting as a preconditioning stimulus which could explain its protective role against ischemic, hypoxic, and toxic damage.

## Abbreviations

ALT: Alanine aminotransferase
AOPP: Advanced oxidation protein products
GSH: Reduced glutathione
GSH-R: Glutathione reductase
GSSG: Oxidized glutathione
HNE: 4-Hydroxynonenal
IPRL: Isolated perfused rat livers
MDA: Malondialdehyde
NO: Nitric oxide
TBARS: Thiobarbituric reactive substances
UPS: Ubiquitin proteasome system.
